# A New Insight Into Missing Data in Intensive Care Unit Patient Profiles: Observational Study

**DOI:** 10.2196/11605

**Published:** 2019-01-08

**Authors:** Anis Sharafoddini, Joel A Dubin, David M Maslove, Joon Lee

**Affiliations:** 1 Health Data Science Lab School of Public Health and Health Systems University of Waterloo Waterloo, ON Canada; 2 Department of Statistics and Actuarial Science University of Waterloo Waterloo, ON Canada; 3 Department of Critical Care Medicine Queen's University Kingston, ON Canada; 4 Department of Community Health Sciences Cumming School of Medicine University of Calgary Calgary, AB Canada; 5 Department of Cardiac Sciences Cumming School of Medicine University of Calgary Calgary, AB Canada

**Keywords:** electronic health records, clinical laboratory tests, machine learning, hospital mortality

## Abstract

**Background:**

The data missing from patient profiles in intensive care units (ICUs) are substantial and unavoidable. However, this incompleteness is not always random or because of imperfections in the data collection process.

**Objective:**

This study aimed to investigate the potential hidden information in data missing from electronic health records (EHRs) in an ICU and examine whether the presence or missingness of a variable itself can convey information about the patient health status.

**Methods:**

Daily retrieval of laboratory test (LT) measurements from the Medical Information Mart for Intensive Care III database was set as our reference for defining complete patient profiles. *Missingness indicators* were introduced as a way of representing presence or absence of the LTs in a patient profile. Thereafter, various feature selection methods (filter and embedded feature selection methods) were used to examine the predictive power of missingness indicators. Finally, a set of well-known prediction models (logistic regression [LR], decision tree, and random forest) were used to evaluate whether the absence status itself of a variable recording can provide predictive power. We also examined the utility of missingness indicators in improving predictive performance when used with observed laboratory measurements as model input. The outcome of interest was in-hospital mortality and mortality at 30 days after ICU discharge.

**Results:**

Regardless of mortality type or ICU day, more than 40% of the predictors selected by feature selection methods were missingness indicators. Notably, employing missingness indicators as the only predictors achieved reasonable mortality prediction on all days and for all mortality types (for instance, in 30-day mortality prediction with LR, we achieved area under the curve of the receiver operating characteristic [AUROC] of 0.6836±0.012). Including indicators with observed measurements in the prediction models also improved the AUROC; the maximum improvement was 0.0426. Indicators also improved the AUROC for Simplified Acute Physiology Score II model—a well-known ICU severity of illness score—confirming the additive information of the indicators (AUROC of 0.8045±0.0109 for 30-day mortality prediction for LR).

**Conclusions:**

Our study demonstrated that the presence or absence of LT measurements is informative and can be considered a potential predictor of in-hospital and 30-day mortality. The comparative analysis of prediction models also showed statistically significant prediction improvement when indicators were included. Moreover, missing data might reflect the opinions of examining clinicians. Therefore, the absence of measurements can be informative in ICUs and has predictive power beyond the measured data themselves. This initial case study shows promise for more in-depth analysis of missing data and its informativeness in ICUs. Future studies are needed to generalize these results.

## Introduction

### Background

The increased adoption of electronic health record (EHR) systems has boosted interest in the secondary use of EHR data [1]. Although the literature has introduced various dimensions for EHR data quality, completeness and correctness have been reported as the fundamental dimensions [1,2]. Although these issues can also be observed in paper-based records, EHR brought us the opportunity to identify them faster and helped us with addressing them. The data missing from clinical contexts are substantial [3,4] and unavoidable [5]; many studies have focused on resolving this issue [6-8]. Although many researchers treat missing data as a challenge [9-18], others continue to debate whether lack of completeness also provides useful information [4,19-21]. Researchers do agree that a part of this incompleteness is not random or because of imperfections in the data collection process [21,22]. Recently, Angiel et al [21] demonstrated that the laboratory ordering time (ie, the interval between 2 orders of a laboratory test; LT) for some LT is more informative than the actual values in predicting 3-year survival. Our study focuses on systematically investigating the implications or possible value of lack of data, particularly in intensive care units (ICUs) and proposes a representation method for missing data to capture hidden information. In general, 2 reasons are given for missing data in EHRs:

- No intention to collect: the clinical variable was never measured because there was no clinical indication to do so—the patient was not suffering from a relevant symptom or comorbidity [4] or it could not be measured [19].
- Intention to collect: records are missing although the variables were measured [4].

Therefore, the health care process (eg, clinicians’ decision to order a test and nurse data entry) affects the recorded EHR and can cause incompleteness in data.

Incomplete EHR data can complicate or prohibit the data analysis process, as many machine learning (ML) algorithms assume that there are no missing data in the dataset or require users to clean the data in the preprocessing stage and so provide a complete dataset. Therefore, from a research perspective, the ideal situation is to increase the amount and accuracy of EHR documentation by employing approaches that focus on intention to collect such as reducing the error in data entry or increasing data documentation in terms of resolution. Although the current amount of testing and bloodwork has been reported as actually redundant in ICUs [23-25] and requires extra time and work from clinicians [4], these approaches suffer from their own shortcomings. Besides analytical methods that can handle missing data (that are missing at random) such as decision trees (DTs) or mixed-effects models for longitudinal data, other approaches usually assume missing data are missing completely at random. In general, the literature proposes 3 analytical approaches: complete case analysis (CCA) or deletion, available case analysis (ACA), and imputation.

CCA starts with the list of variables included in the analysis and discards records with missing data on any of the variables. However, this subsample might not be a random sample of the population. Although researchers argue that sample selection based on the predefined eligibility criteria in randomized clinical trials can limit the external generalizability of these studies [26], CCA in studies using EHR data can also potentially threaten the external validity of a study [19] and cause bias as the literature shows a statistically significant relationship between severity of illness and data completeness [20]. A study [19] on 10,000 EHRs from patients receiving anesthetic service showed that patients with an anesthesiologists physical status (ASA) [27] class-4 fitness rating had 5.05 more days with laboratory results and 6.85 more days with medication orders than patients with ASA class 1, suggesting more data are recorded for sicker patients than healthier patients. Thus, imposing complete case requirements when using EHR data for secondary use can cause bias toward selecting patients with more severe conditions (or several comorbidities). Despite this drawback, CCA has been identified as the leading approach in studies on ICU data [28]. That said, CCA provides valid inference only when data are missing completely at random (MCAR), which is unlikely in practice [29].

The ACA (or pairwise deletion) uses all available data for a given analysis. In other words, it maximizes the availability of data by an analysis-by-analysis basis [30]. The advantage of this method is that more data are included in each analysis than with CCA. It also allows for valid inference by likelihood-based models when missing data are ignorable—often the case when the data are missing at random (MAR) [29]. Although ACA is an improvement to CCA [30], it also has limitations. As different samples are being used in each analysis, not only is comparison of various analyses impossible [31] but also using different samples for estimating the parameters of interest has occasionally led to biased or mathematically inconsistent results [32-34].

Imputation methods, which try to draw inferences from incomplete data, rely on knowing the mechanism of missingness, which cannot be validated from the available data. Single imputation methods suffer from 2 problems. First, an inference based on imputed data can be biased if the underlying assumptions are not valid. Second, because imputed data are assumed to be true, the model’s statistical precision is overstated. Multiple imputation methods, in spite of their promising performance, rely on parametric assumptions that, if not valid, can lead to incorrect imputation. Due to these limitations, imputation methods should be used with caution and checking underlying assumptions with clinicians is highly recommended [5]. However, Gorelick [35], in a simulation study, demonstrated that either CCA or imputation could cause bias in predictive modeling, and that assuming missing values to be normal when missingness rates are high and substituting them with normal values would also cause substantial bias. In brief, if primary assumptions are not fully satisfied, neither considering complete or available cases nor imputating missing data is likely to yield reliable results. Furthermore, these statistical methods on their own are not sufficient to capture the hidden information about the patient health status and care process in the complex EHR data. Alternatively, we can try to learn from what is missing rather than only dealing with missingness as a deficiency.

### Objectives

This case study provides evidence that missing data in ICU might be missing because of the patient’s health status or health care process and introduces a new method for representing patient profiles. In this representation, auxiliary variables, called indicators, are used to represent the presence or absence of a measurement and might convey the possible hidden information in the missing data. Then, by employing various analytical methods, this study attempts to demonstrate the informativeness of missing data. In the rest of the study, the term *missing data* is used to describe not-at-random missing information in patient profiles. In other words, the potential informativeness of data that has not been recorded by choice is of interest.

## Methods

### Measurement Protocol and Data Collection

As patient monitoring strongly relies on clinical needs, no universal standards for ICU data completeness have been established [36-38]. However, a study by Frassica in 2005 [39] published a list of the top 80% of LTs common to all ICU patients within a university teaching hospital. We revised this list based on the presence of these tests in our database and updated it with input from an ICU clinician to reflect current practices (Textbox 1).

The data for this study were collected from the Medical Information Mart for Intensive Care III (MIMIC-III) [40] database which contains data from 38,597 distinct adult patients admitted to the Beth Israel Deaconess Medical Center in Boston, Massachusetts, between 2001 and 2012. For patient cohort selection, a tailored version of the generalized cohort selection heuristics for retrospective EHR studies introduced by Harrell et al [41] was used. The data for first admission to 1 of the 5 ICUs—medical ICU, surgical ICU, cardiac care unit, cardiac surgery recovery unit, and trauma surgical ICU—were extracted for adult patients (aged 15 years or older). Included patients must have had at least one data point in any of the variable categories during the first, second, and third days of their ICU stay.

### Data Preprocessing and Missing Data Representation

Each day’s extracted data were mapped into a matrix with columns for measurements and rows for patients. Therefore, we had a column for each daily measurement of LTs, resulting in 36 columns for LTs. An auxiliary matrix was generated to store binary values reflecting the presence (0) or absence (1) of measurements. As many well-performing ML algorithms are designed to work with a complete data matrix, 2 methods—predictive mean matching (PMM) [42] and hot deck (HD)—were used to impute missing values. PMM is a commonly used and well-accepted imputation method in public health research [43] and is also robust against model misspecification [44]. HD imputation is used commonly in applied data analysis when missing data exist [45].

**A total of 36 laboratory tests used in investigating informativeness of missing data.**

Variable category and variables

Top 80% laboratory tests and profiles common to all intensive care units [39] reviewed and revised by domain expert

- Alanine aminotransferase (ALT)
- Alkaline phosphatase (ALK)
- Aspartate aminotransferase (AST)
- Arterial blood gases: pH, partial pressure of carbon dioxide (PCO_2_), and partial pressure of oxygen (PO_2_)
- Base excess (BE)
- Basic metabolic panel: sodium (Na), potassium (K), chloride (Cl), bicarbonate (HCO_3_), anion gap (AG), blood glucose (BG), blood urea nitrogen (BUN), and creatinine (Cr)
- Complete blood count: white blood cells (WBCs), red blood cells (RBCs), hemoglobin (HGB), hematocrit (HCT), mean corpuscular volume (MCV), mean corpuscular hemoglobin (MCH), mean corpuscular hemoglobin concentration (MCHC), red cell distribution width (RDW), platelet count (PLT), absolute monocytes (MO), absolute eosinophils (EO), absolute basophils (BA), absolute lymphocytes (LY), and absolute neutrophils (NE)
- Lactate (Lac)
- Calcium (Ca)
- Magnesium (Mg)
- Phosphate (Phos)
- Partial thromboplastin time (PTT)
- Prothrombin time (PT)
- Total bilirubin (TBil)

**Figure 1:**
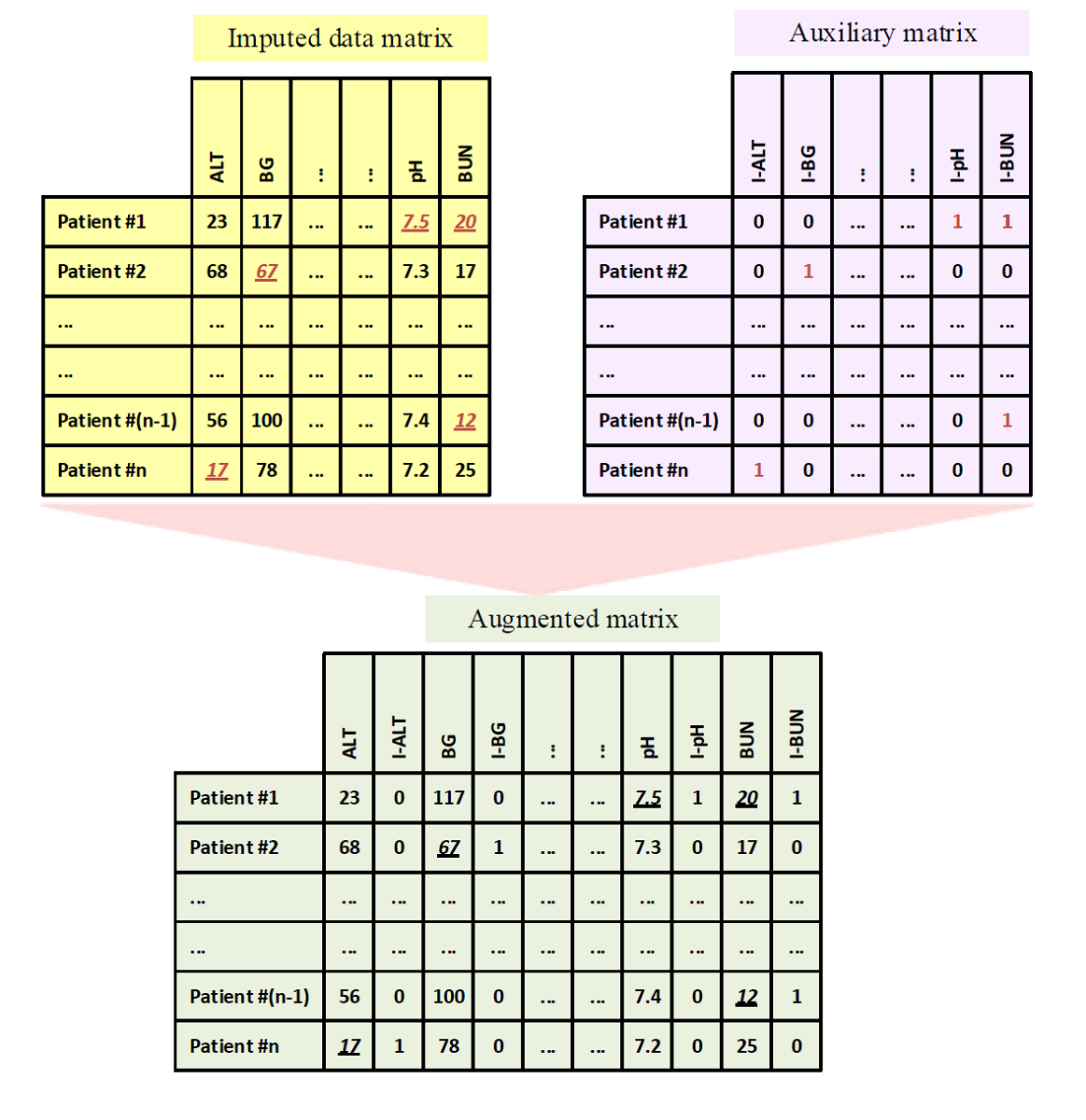
An example of the augmented data matrix, the imputed data matrix (imputed values are underlined and italicized), and the auxiliary matrix (containing the missingness indicators: 0-present, 1-absent).

Given that imputed values are indistinguishable to the ML algorithm from true values, we combined the original matrix and auxiliary matrix to form an augmented matrix that directly indicates where values were imputed. This was done to mitigate the risk of treating imputed values the same as actual values, in a setting where the underlying reason for missing data is not fully known (Figure 1). Missing data indicators in this augmented matrix might also provide extra information about the reliability of the values (actual and imputed values) and potentially preserve any meaningful missing data patterns. Missingness indicators have been used as a method of handling missing data in epidemiological and clinical studies. However, in the current use of indicators, missing values are set to a fixed value (0 or the normal value for the variable) and the indicators are used as dummy variables in analytical models to indicate that a value was missing [46,47]. Studies have shown that this method causes bias as the missing values are imputed with a single value [48]. In our study, we are not using indicators as dummy variables; instead, we are introducing them as a source of information to be used besides imputation methods.

### Validation

Several validation techniques are available in medical research. In this study, for all experiments where applicable, we used cross-validation technique (10-fold cross-validation). We also repeated the cross-validation procedure several times (20 times) to acquire more stable results as suggested in the literature [49].

### Assessments

#### Exploratory Analysis

First, the trends of missingness among LTs were visualized for comparison. Afterward, pairwise correlation among indicators, using Phi coefficient, was done to explore the general behavior of missingness. The Elixhauser [50] and the Charlson [51] comorbidity indices are the most common comorbidity scores in clinical applications. The literature has shown that the Elixhauser Comorbidity Index (ECI) in general has the best performance [52-55]. This better performance can be the result of (1) including new comorbidities in ECI, (2) the differences in the coding of variables common between both indices, or (3) a combination of the first and second factors [53]. The Simplified Acute Physiology Score II (SAPS-II) [56] scoring system that has been widely used by most ICUs for predicting illness severity was also chosen. Therefore, the association of missingness rates with ECI and SAPS-II was investigated using Spearman correlation. Besides the clinical information, SAPS-II also has the information about type of admission (scheduled surgical, medical, or unscheduled surgical) and presence of 3 chronic diseases (metastatic cancer, hematologic malignancy, and AIDS).

#### Feature Selection

After exploratory analyses, we assessed the importance of the indicators as potential predictors. First, we used feature selection methods, which are widely used to determine which predictors should be used in a model, particularly for high-dimensional data [22]. Two copies of the augmented matrix (derived from HD and PMM imputation) were fed to various feature selection methods. Our study considered in-hospital and 30-day postdischarge mortality as outcomes. Overall, we used 2 categories of supervised feature selection methods described below.

First, filter techniques evaluated the importance of a predictor by looking at data properties. Filter methods, in general, use a metric to identify irrelevant features and filter out the redundant predictors from the data matrix [57]. We selected 3 different metrices: LR beta value, relief algorithm [58], and information gain (InfGain) [59]. The relief algorithm examines the relevance of predictors based on their power to distinguish between similar patients with the same and different outcome. InfGain measures the reduction in entropy of the class variable achieved by partitioning the data based on the index predictor; relevant predictors receive a high InfGain value [60]. This ensemble of the scoring methods was then used to determine the normalized informativeness of all predictors. Aggregating these methods in one score provides a tool for comparing predictors from different aspects.

Second, we used embedded techniques to search for the optimal set of predictors. In these techniques, feature selection is embedded in the model’s construction and interacts with the classifier. Least absolute shrinkage and selection operator (LASSO), used in this study, is a penalizing method in this category. LASSO regression in its objective functions considers a penalty that equals to the sum of the absolute values of the coefficients. As absolute function (L_1_ norm) is not differentiable, the estimated coefficients are close to 0, and some will be exactly 0 resulting in an automatic variable selection. For this and the next experiments, 10-fold cross-validation with 20 repeats was used (leading to 200 repetitions in total). This number of repetitions is recommended to achieve desired accuracy for prediction performance estimation [49].

#### Predictive Modeling

In the last assessment, we first trained group of classification models, including DT, logistic regression (LR), and random forest (RF), on the indicator and imputed data matrices and evaluate their performance for predicting desired outcomes using the area under the curve of the receiver operating characteristic (AUROC) validation metric. Thereafter, new models were trained using the augmented data matrix and their performance was compared with that of the original to determine whether the indicators have predictive power and can boost the models’ predictive accuracy. We also investigated the predictive performance of SAPS-II score, and then we added indicators to these scores to examine the impact of indicators beyond SAPS-II score. It is worth mentioning that in this assessment, the absolute accuracy of the models is not of our interest, instead, the relative improvement in the performance when including indicators as input. That is, achieving the best possible mortality prediction AUROC is not the objective of this study.

## Results

### Population

**Figure 2:**
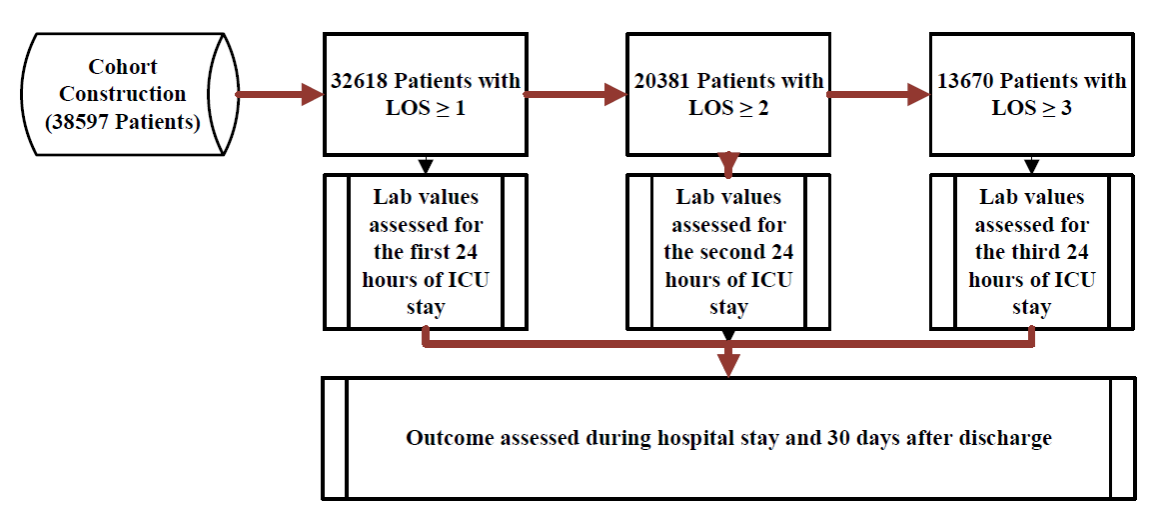
The retrospective cohort study design. LOS: length of stay.

The analyses of the first 24 hours ICU stays included 32,618 patients but decreased to 20,381 for the second 24-hour interval, as many patients were discharged after 24 hours. The third 24-hour period included 13,670 patients. Of these groups, 10.99% (3586/32,618), 13.59% (2769/20,381), and 16.19% (2213/13,670) experienced death in-hospital and 15.12% (4933/32,618), 18.26% (3722/20,381), and 21.32% (2915/ 13,670) experienced death within 30 days of discharge, respectively. Figure 2 demonstrates the retrospective study design.

### Exploratory Analysis

**Figure 3:**
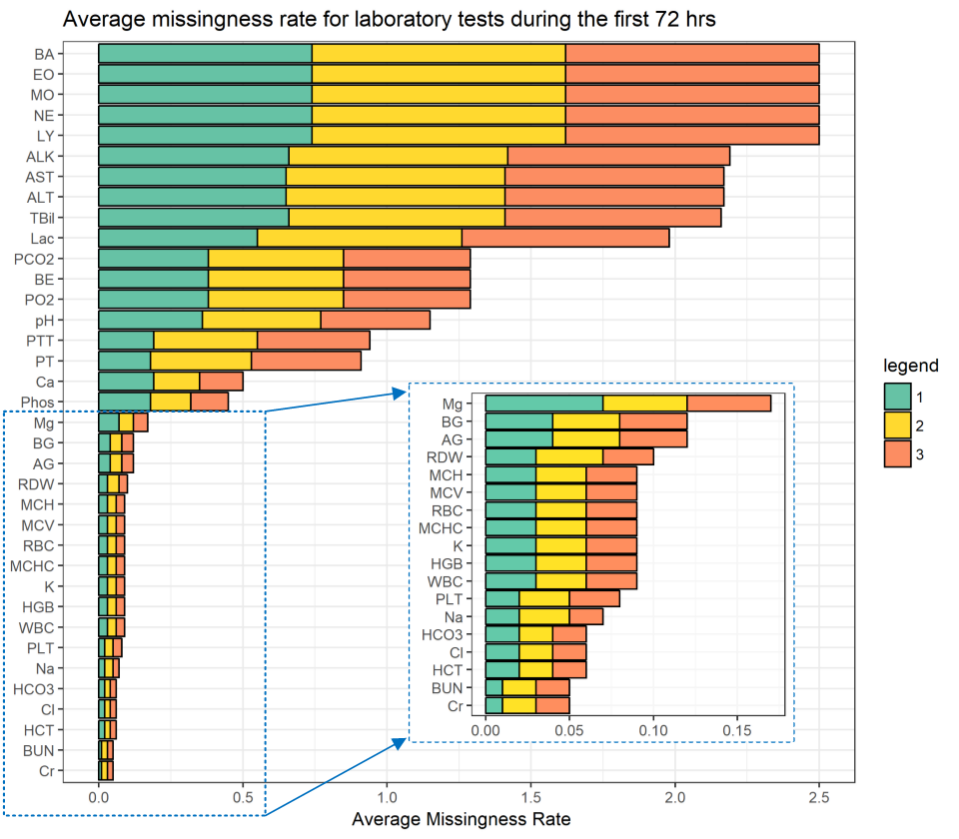
The average missingness rate among patients for laboratory tests in the first 72 hours of admission.

Missingness rates for LTs ranges from 1.36% (445/32,618) to 88.27% (12066/13,670) in the first 72 hours after admission. Figure 3 shows the missingness rate for LTs over 3 days. Absolute basophils (BA), absolute eosinophils (EO), absolute monocytes (MO), absolute lymphocytes (LY), absolute neutrophils (NE), alanine aminotransferase (ALT), alkaline phosphatase (ALK), aspartate aminotransferase (AST), total bilirubin (TBil), and lactate (Lac) were among the less-common LTs and were missing in the profiles of more than 60% of patients.

We calculated the association between each indicator and the mortality flag. Although association values were small, on day 1, ALT, ALK, AST, and TBil stand out as the top LTs associated with both types of mortality.

On days 2 and 3, partial pressure of carbon dioxide (PCO_2_), partial pressure of oxygen (PO_2_), and base excess (BE) were the top LTs associated with both mortality types. Lac also joined the top tests on day 2 for 30-day mortality. Detailed association values are provided in See Multimedia Appendix 1.

**Figure 4:**
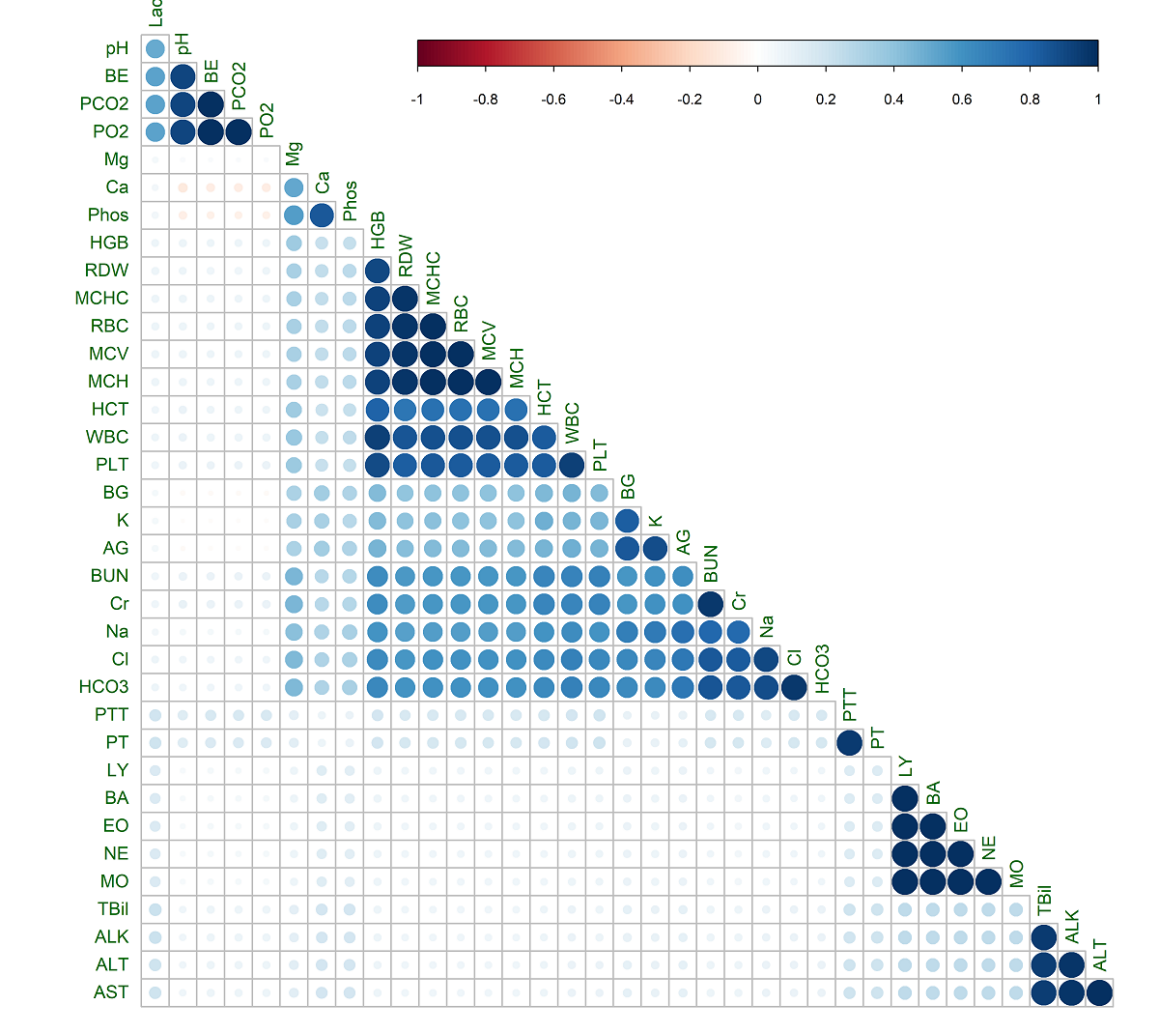
Visualization of the correlation matrix for variable indicators in first 72 hours.

Figure 4 visualizes the pairwise correlations among indicators. In total, 7 major groups of highly correlated (ρ ≥.95) indicators were observed in the results using Phi coefficient: (1) BA, MO, NE, EO, and LY; (2) mean corpuscular hemoglobin concentration (MCHC), red cell distribution width (RDW) mean corpuscular volume (MCV), red blood cell (RBC), and mean corpuscular hemoglobin (MCH); (3) BE, PCO_2_, and PO_2_; (4) TBil, ALT, AST, and ALK; (5) Blood urea nitrogen (BUN) and creatinine (Cr); (6) chloride (Cl) and bicarbonate (HCO_3_); (7) partial thromboplastin time (PTT) and prothrombin time (PT).

The Spearman correlation between missingness rates and ECI was also calculated daily. Results show a statistically significant correlation between these variables (day 1: ρ=–.233; day 2: ρ=–.196; day 3: ρ=–.184; *P*<.001). The same assessment was done using SAPS-II. The results were in line with the previous one and demonstrate higher correlation (day 1: ρ=–.315; day 2: ρ=–.277; day 3=–.234; *P*<.001). These findings are interesting as they confirm that the missingness of data is associated with patient severity of illness.

### Feature Selection: Missing Data Indicators as Important Predictors

Each of the imputation methods was applied to the original dataset, and the potential informativeness of missingness indicators in comparison with actual variables was investigated using an ensemble of the most representative filter selection methods [61]: LR beta value, relief, and InfGain. Table 1 shows the top 18 variables selected on each day based on the PMM-generated imputed matrix predicting 30-day mortality. BUN, RDW, and anion gap (AG) were among the top variables in all 3 days. Indicators for TBil, phosphate (Phos), calcium (Ca), and Lac were selected on the first day, whereas indicators for Lac, BE, PO_2_, and PCO_2_ were among the top features on the second and third days. PTT and pH indicators were also among the important indicators on the third day.

**Table 1:** The top 18 variables selected on each day after employing predictive mean matching imputation with regard to 30-day mortality. *I* at the beginning of the variables’ names means *indicator*. Numbers represent the ranking after aggregating the ranking results from the 3 different feature selection methods.

Day 1		Day 2		Day 3	
Variable	Score	Variable	Score	Variable	Score
BUN^a^	.762397	AG^b^	.795419	RDW^c^	.748997
RDW	.680087	HCO_3_^d^	.783337	BUN	.666667
MCHC^e^	.668965	BUN	.77677	HCO_3_	.544964
AG	.540484	BE^f^	.609532	BE	.540542
I-Ca^g^	.436429	RDW	.608711	pH	.488433
Cr^h^	.436071	I-PO_2_^i^	.587151	AG	.450426
HCO_3_	.416741	I-PCO_2_	.585947	I-Lac^j^	.418716
PO_2_^k^	.404289	I-BE	.585592	I-pH	.40463
MCV^l^	.386964	Cl^m^	.53158	Cr	.400008
I-Phos^n^	.374431	PT^o^	.462085	Phos	.387661
PTT^p^	.353913	Lac	.461869	I-PCO_2_	.387019
HGB^q^	.342786	Cr	.451999	I-PO_2_	.386739
pH	.32767	PTT	.424956	I-BE	.385935
Lac	.320339	Na^r^	.422474	PCO_2_	.367257
BE	.320299	Phos	.419171	NE^s^	.360791
I-Lac	.318216	I-Lac	.415475	MCV	.351266
PCO_2_	.316668	MCV	.368343	I-PTT	.338352
I-TBil^t^	.31277	MCHC	.363146	Lac	.331205

^a^BUN: blood urea nitrogen.

^b^AG: anion gap.

^c^RDW: red cell distribution width.

^d^HCO_3_: bicarbonate.

^e^MCHC: mean corpuscular hemoglobin concentration.

^f^BE: base excess.

^g^CA: calcium.

^h^Cr: creatinine.

^i^PO_2_: partial pressure of oxygen.

^j^Lac: lactate.

^k^PCO_2_: partial pressure of carbon dioxide.

^l^MCV: mean corpuscular volume.

^m^Cl: chloride.

^n^Phos: phosphate.

^o^PT: prothrombin time.

^p^PTT: partial thromboplastin time.

^q^HGB: hemoglobin.

^r^Na: sodium.

^s^NE: absolute neutrophils.

^t^TBil: total bilirubin.

Similar results were observed when using the HD imputation method, except that ALT and Phos were also selected on the first and second day, respectively. Moreover, PTT and pH indicators were not among the important indicators on the third day. Detailed results of this assessment can be found in Multimedia Appendix 1.

Results for in-hospital mortality were slightly different (Table 2). Although the selected indicators were almost the same as for 30-day mortality, more indicators were selected on the first day for in-hospital mortality, implying that indicators are more associated with in-hospital mortality than 30-day mortality. Detailed results are available in Multimedia Appendix 1.

To validate our previous results, we assessed the predictive power of the indicators using embedded feature selection methods. Each day, a LASSO model was trained on the augmented data from HD and PMM imputation using 10-fold cross-validation with 20 repeats. In general, the AUROC of mortality prediction (in-hospital and 30-day postdischarge) and number of selected variables decreased from days 1 to 3 (Table 3).

Moreover, prediction of in-hospital mortality resulted in higher AUROCs than 30-day mortality. Regardless of mortality type, on all days, more than 40% of the predictors selected by the best-performing model were indicators. Moreover, more than 61% of selected predictors were indicators on the third day. Sliding lambda to compromise the predictor number and model performance led to almost the same results. Generally, more than 40% of the selected predictors were indicators, and on the third day, this number increased to 61%.

Results in this section once more confirm the informativeness of missing data as missingness indicators have been selected by various feature selection methods. The high percentage of selected indicators also implies that the actual value of an LT is not always required in outcome prediction; instead, knowledge about whether the test was performed would suffice.

### Predictive Modeling: Missing Data Indicators in Predictive Modeling

**Figure 5:**
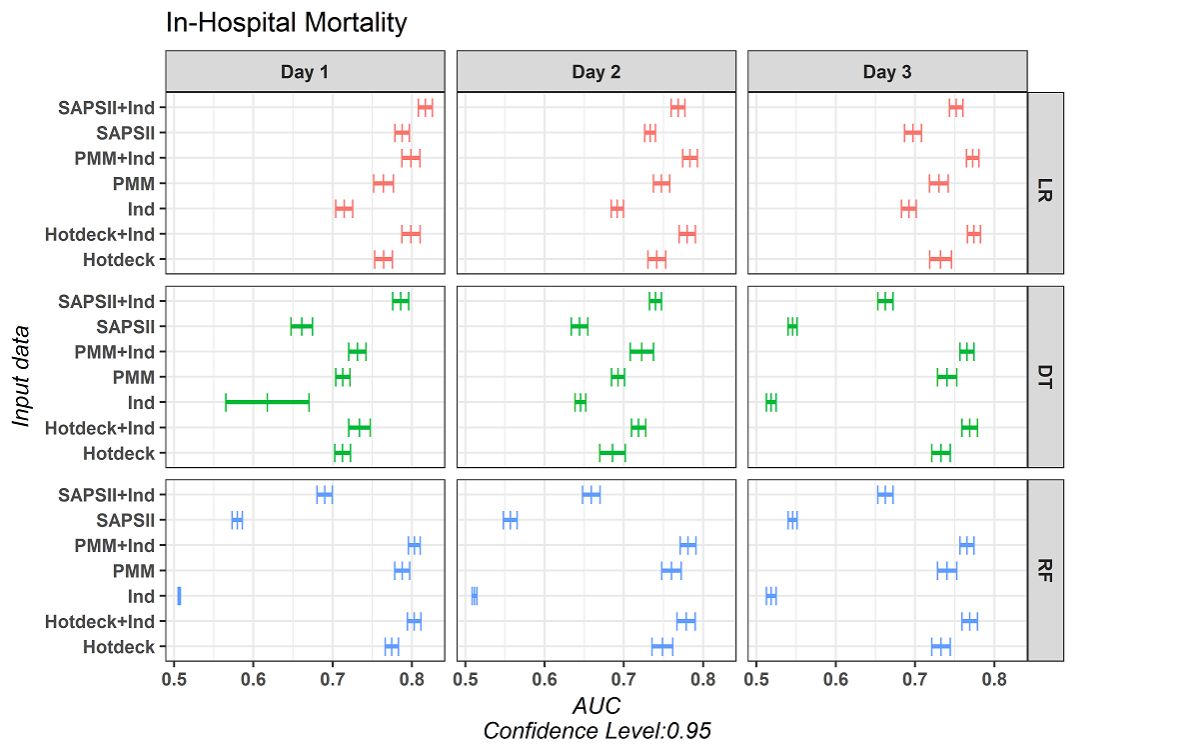
The 95% CIs of the area under the curve of the receiver operating characteristic for logistic regression, decision tree, and random forest models on missingness indicators, simplified acute physiology score-II, and actual variables with and without the missingness indicators.

In the second assessment, we compared the performance of a set of 3 classification models (DT, LR, and RF) using the indicators, imputed and augmented data matrices, and SAPS-II score with or without indicators with 10-fold cross-validation over 20 repeats. We investigated whether including indicators can improve prediction and whether indicators alone have predictive power. For our LR, the iteratively reweighted least square method was used to fit the model. The complexity parameter (CP) for DT was tuned based on the model performance. On the basis of some preliminary model fitting, we set the CP value to vary from 0 (including all variables and having a large tree) to .02 for each model and then we picked the best performance model. In all models, the best-tuned model had a CP greater than 0. Figure 5 shows the AUROC with 95% CI for all 3 days with regard to 30-day mortality (Multimedia Appendix 1 provides the AUROC values for 30-day mortality and in-hospital mortality).

Including indicators improved the AUROC in all modeling techniques, on average by 0.0511; the maximum improvement was 0.1209 (Figure 5). AUROC has been demonstrated as an insensitive metric, for which an increase of 0.01 suggests meaningful improvement and is clinically of interest [62-64]. Although using only indicators demonstrated reasonable performance in all scenarios (AUROC=0.6019 [0.0862]>0.5), conventional scores such as SAPS II perform better (AUROC=0.6390 [0.0853]) on their own. Therefore, models trained only on indicators are not sufficient. However, including indicators with conventional scores can improve the performance (AUROC=0.7263 [0.0578]). The SAPS-II score has information for age, heart rate, systolic blood pressure, Glasgow coma scale, temperature, mechanical ventilation administration, partial pressure of oxygen in the arterial blood (PaO_2_), fraction of inspired oxygen (FiO_2_), urine output, BUN, sodium (Na), potassium (K), HCO_3_, TBil, white blood cells (WBCs), presence of chronic diseases, and type of admission. These results demonstrate that indicators have information beyond that included in SAPS-II.

**Figure 6:**
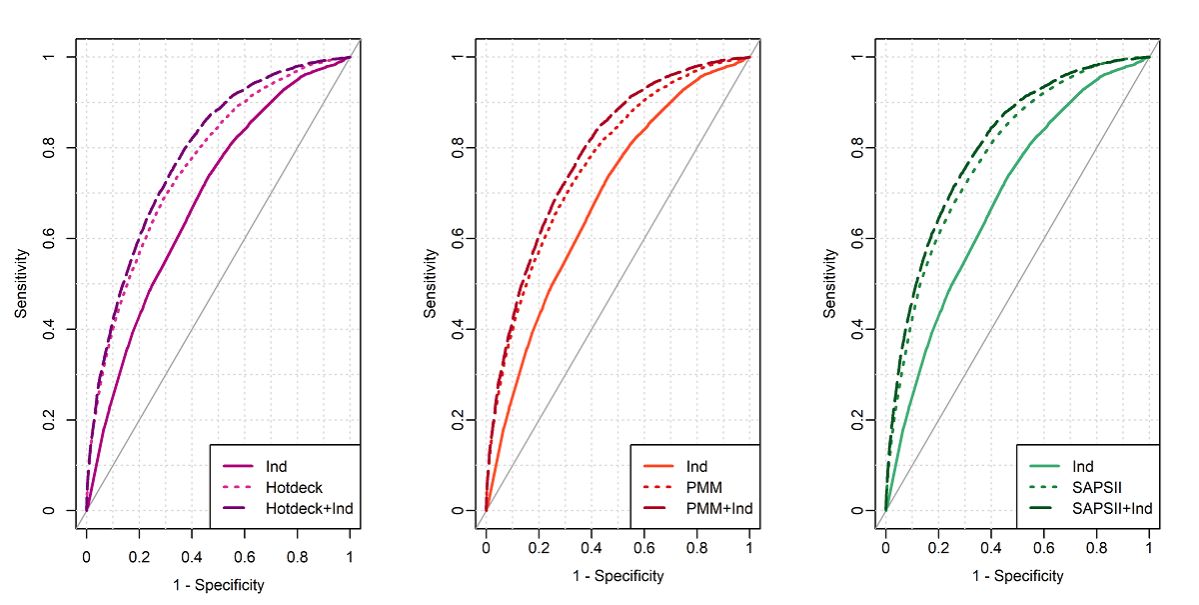
The receiver operating characteristic curves for logistic regression 30-day mortality prediction on day 1.

Figure 6 demonstrates the AUROC curves for LR 30-day mortality prediction on day 1.

This combination of findings provides more support for the informativeness of missing data. Employing the missing indicators in mortality prediction modeling can improve the results in comparison to not including them.

**Table 2:** The top 18 variables selected on each day after employing predictive mean matching imputation with regard to in-hospital mortality. *I* at the beginning of the variables names means *indicator*. Numbers represent the ranking after aggregating the ranking results from the 3 different feature selection methods.

Day 1		Day 2		Day 3	
Variable	Score	Variable	Score	Variable	Score
BUN^a^	.825715	BUN	1	RDW^b^	.75246
AG^c^	.668918	RDW	.711852	BUN	.635729
RDW	.573188	HCO_3_^d^	.684191	BE^e^	.633926
HCO_3_	.531746	AG	.664339	HCO_3_	.62367
MCHC^f^	.507343	BE	.528778	I-BE	.595553
PCO_2_^g^	.489483	MCHC	.503805	I-PCO_2_	.595238
Cr^h^	.480181	PT^i^	.453111	I-PO_2_^j^	.594924
BE	.452599	Cl^k^	.429405	pH	.556242
I-Lac^l^	.436382	I-Lac	.425279	Phos^m^	.494694
Lac	.415773	Cr	.395266	AG	.492864
HGB^n^	.414263	I-PO_2_	.382404	I-pH	.470007
pH	.402466	I-PCO_2_	.381737	I-Lac	.469215
I-TBil^o^	.399363	I-BE	.381448	Cr	.415249
I-Ca	.395278	PTT^p^	.357339	Lac	.396136
I-ALT^q^	.376004	Phos	.352738	NE^r^	.338372
I-AST^s^	.375944	Na^t^	.345109	PT	.326491
LY^u^	.375163	I-PT	.333936	LY	.319146
I-ALK^v^	.366346	BG^w^	.320947	MCV^x^	.314868

^a^BUN: blood urea nitrogen.

^b^RDW: red cell distribution width.

^c^AG: anion gap.

^d^HCO_3_: bicarbonate.

^e^BE: base excess.

^f^MCHC: mean corpuscular hemoglobin concentration.

^g^PCO_2_: partial pressure of carbon dioxide.

^h^Cr: creatinine.

^i^PT: prothrombin time.

^j^PO_2_: partial pressure of oxygen.

^k^Cl: chloride.

^l^Lac: lactate.

^m^Phos: phosphate.

^n^HGB: hemoglobin.

^o^TBil: total bilirubin.

^p^PTT: partial prothrombin time.

^q^ALT: alanine transaminase.

^r^NE: absolute neutrophils.

^s^AST: aspartate transaminase

^t^Na: sodium

^u^LY: absolute lymphocytes.

^v^ALK: alkaline phosphatase.

^w^BG: blood glucose.

^x^MCV: mean corpuscular volume.

**Table 3:** Results from feature selection by least absolute shrinkage and selection operator (LASSO) for 3 days (area under the curve of the receiver operating characteristics are reported with the SE). The *best performing model* refers to the model with a lambda value associated with minimum cross-validation error. The adjusted model refers to a LASSO model with the largest value of lambda such that the error remains within 1 SE of the minimum.

Criteria, outcome, and imputation method			Day 1	Day 2	Day 3
**AUROC^a^** **for best performing model**					
	**30-day mortality**				
		HD^b^	0.7858 (0.0033)	0.7685 (0.0041)	0.7302 (0.0043)
		PMM^c^	0.7876 (0.0039)	0.7708 (0.0046)	0.7391 (0.0053)
	**In-hospital mortality**				
		HD	0.7983 (0.0040)	0.7804 (0.0046)	0.7476 (0.0042)
		PMM	0.8007 (0.0047)	0.7838 (0.0049)	0.7582 (0.0054)
**Indicators among selected predictors by the best performing model, n (%)**					
	**30-day mortality**				
		HD	23 (43)	24 (48)	19 (707)
		PMM	26 (45)	26 (47)	17 (68)
	**In-hospital mortality**				
		HD	28 (46)	29 (48)	21 (60)
		PMM	29 (47)	27 (49)	24 (62)
**AUROC for adjusted model**					
	**30-day mortality**				
		HD	0.7826 (0.0034)	0.7646 (0.0043)	0.7262 (0.0041)
		PMM	0.7840 (0.0038)	0.7667 (0.0045)	0.7339 (0.0044)
	**In-hospital mortality**				
		HD	0.7944 (0.0043)	0.7762 (0.0047)	0.7439 (0.0041)
		PMM	0.7961 (0.0049)	0.7793 (0.0050)	0.7536 (0.0045)
**Indicators among selected predictors by the adjusted model, n (%)**					
	**30-day mortality**				
		HD	20 (45)	16 (48)	22 (67)
		PMM	19 (45)	16 (52)	31 (62)
	**In-hospital mortality**				
		HD	20 (47)	13 (42)	16 (64)
		PMM	18 (50)	11 (41)	16 (62)

^a^AUROC: area under the curve of the receiver operating characteristic.

^b^HD: hot deck.

^c^PMM: predictive mean matching.

## Discussion

### Principal Findings

We used missingness indicators to represent missing information in patient profiles in ICU. The informativeness of these indicators was demonstrated in 3 sets of assessments. First, our exploratory analysis confirms that the missingness of data is associated with patient severity of illness or comorbidities. Afterward, by means of feature selection methods, the predictive power of the presence of an LT in the patient profile was found to be more than the actual measured value. Finally, missingness indicators noticeably improved the performance of mortality prediction models. The high correlation observed among some of the variable indicators suggests that all the variables in a set are typically measured or ordered together. Therefore, if a patient is missing 1 variable of a set, he or she will likely be missing the others as well. This fact is well represented in all 7 groups. The first group comprises the differential WBC counts (BA, MO, NE, eosinophil; EO, and LY), which itemizes the number of basophils, monocytes, neutrophils, eosinophils, and lymphocytes among present WBCs. The second group (RDW, MCHC, MCV, RBC, and MCH) comprises tests that are used to measure the actual number of RBCs and their physical characteristics. The third group (BE, PCO_2_, and PO_2_) consists of blood gas components and focuses on oxygen and carbon dioxide pressure as well as excess or deficit of base levels in the blood. Tbil, ALT, AST, and ALK in the fourth group are liver enzymes [65] that are ordered when a patient is suffering from or showing symptoms of a liver-related comorbidity. BUN and Cr mainly focus on kidney function. Bicarbonate; HCO_3_ and chloride; Cl are the primary measured anions in the blood. PT along with PTT are used for investigating hemostasis and are the starting points for looking into potential bleeding or clotting complications. Therefore, the presence of a clinical variable in a patient profile can represent a comorbidity in the patient. Although LTs are mainly ordered for diagnostic and prognostic reasons, studies have shown widely diverse test-ordering behavior among clinicians for similar symptoms [66-68]. Therefore, indicators could also reflect the opinions, preconceptions, and biases of the treating clinicians. In other words, by using the missingness indicators, we are learning from practice patterns rather than physiologic patterns. Therefore, indicators as introduced in this study can then be used for modeling health care process in various applications such as clinical care, clinical research, health care economics, and health care policy [21,69].

Filter methods verified the importance of some indicators with regard to our outcomes. Results also demonstrated that indicators become more and more important on ICU days 2 and 3 (Tables 1 and 2). This observation aligns with clinical practice in which ICU clinicians might try to get a complete dataset on day 1 to fully investigate the patient and understand the situation but are likely to be more selective with LT ordering on subsequent days. The Lac indicator was associated with 30-day and in-hospital mortality on the second and third day. Lactate is usually used as a biomarker for shock states. The literature has constantly reported an association between lactate levels and mortality rates among critically ill patients [70]. Our study demonstrated that just the presence of this information could represent the severity of a patient’s illness, as patients with profound shock have a very high mortality rate in hospitals and ICUs [71]. Moreover, BUN [72-74], RDW [75-79], and AG [80-83] have been repeatedly determined as a risk factor of all-cause mortality and their indicators received a high score in our analysis. These results are consistent with those of Agniel et al’s [21] who demonstrated that the presence of these tests have significant association with odds of 3-years survival.

The LASSO model selected indicators among the clinical predictors of in-hospital mortality and 30-day mortality, implying the predictive power of indicators. More indicators than clinical variables were selected on the third day (60%-70% of selected predictors were indicators); the assessment demonstrates that indicators from the third day are more informative than those from the first, again supporting the idea that the practice patterns diverge later during ICU stays, so there is more variability in what gets measured. In other words, care on the first day is likely to be highly protocolized—all patients get the same tests regardless of their condition because their trajectory is still unclear. As time goes on, the patterns become more evident and ordering and prescribing practices change according to clinical need. This high percentage of selected indicators suggests that clinical variables are not always required in outcome prediction; instead, information about their presence would suffice.

The last assessment demonstrated that models trained on indicators alone in some scenarios have reasonable performance (for instance, in 30-day mortality prediction with LR, we achieved AUROC of 0.6836 [0.012]). These results imply that by considering missing data as noise or a random artifact, we can lose valuable information about patient outcomes. Moreover, indicators improved the AUROCs in most scenarios. Researchers in this field are looking for predictors that can be included in the models to improve the prediction results. Having a low-dimensional set of typical predictors plus these missing data indicators can actually lead to performance comparable with that achieved using typical predictors plus other potentially useful predictors identified a priori by medical researchers: First, in comparison with including extra numeric predictors, the computational load for performing mathematical calculations on binary values such as indicators is usually less. Second, binary data require less computational memory than numbers when performing data mining techniques. Finally, for some important clinical variables, storing the missing data indicators instead of the actual value better protects patient privacy while preserving predictive power. In other words, less privacy concern is expected in a situation when the type of test is disclosed rather than the actual test result. The comparative analyses on the predictive models showed that missing data indicators could improve the prediction models’ performance. Although literature considers a small increase (0.01) in AUROC meaningful and of clinical interest (because of insensitivity of AUROC) [62,64], including the indicators in our study could improve the average AUROC by 0.0511. Thus, missing data indicators can be introduced as informative predictors and be used to learn from. In other words, these indicators can be representative of physicians’ and patients’ opinions during the health care process. Furthermore, the overall model performance decreased over time perhaps implying that patients’ data on the first 24-hour has the highest level of information. The same pattern was also observed in the previous assessment. According to these observations, we can infer that presence or absence of a variable can be used in predicting patients’ severity of illness.

### Strengths and Limitations of the Project

A significant strength of this study is its new insight on missing data in a real-world ICU database. The results confirm the predictive power of some indicators and their advantage over actual values in predictive modeling. The findings further clarify the factors associated with lack of data collection such as the healthier status of a patient or practice patterns of clinicians. These insights, in turn, can be used to design models that consider missing data and benefit from the hidden information. On the basis of our results, missingness indicators can be introduced as potential predictors of ICU patients’ outcome.

Despite the strength, significance, and novel nature of this study, there also exist limitations that cannot be overlooked. First, because of the nature of ICUs, the amount of missing data in MIMIC is less than that from a general ward. Therefore, our study may not fully demonstrate the informativeness of these indicators. Moreover, adding the indicators of interest to the actual data matrix increases the dimension of the matrix and may become computationally burdensome. Using other imputation methods, the power of missing data indicators may vary but this was beyond the scope of our study, which focused on providing evidence on missing data informativeness.

### Perspectives for Future Work

Although our study demonstrates that missingness indicators are informative and have predictive power in mortality prediction in ICU, further studies are required to investigate their power in predicting other clinical outcomes. Future researchers can investigate the association between missingness patterns and patient diagnosis. They can also consider more sensitive criteria such as net reclassification or integrated discrimination improvements while preserving improvement in the AUROC as the first criterion. Moreover, as this study looked at the 3 days in the ICU independently, one can investigate if the missing data on a particular day are still informative given all the clinical and indicator variables from previous days. These future studies should also investigate the effect of missing rate on the predictive power of indicators. Another area of future work is examining the test-ordering behavior among clinicians, by using missingness indicators.

### Conclusions

Our study has demonstrated that the missingness of data itself might be informative in ICU and might have added predictive value beyond observed data alone. Moreover, indicators for variables with higher missingness rates had more predictive power. In practice, the lack of a set of symptoms might lead health professionals to conclude that a particular set of tests is not required at the current stage. Therefore, these missing data are not a random occurrence. This study showed that the number of comorbidities is associated with a decreased rate of missing data. Therefore, rudimentary treatments of missing data (eg, CCA) can cause bias toward sicker patients. The study is also notable because it provided new insight about the informativeness of missing data and described how this information could be used in predicting mortality.

## Appendix

Multimedia Appendix 1 Detailed results.

## Abbreviations

ACA: available case analysis
AG: anion gap
ALK: alkaline phosphatase
ALT: alanine aminotransferase
ASA: anesthesiologists physical status
AST: aspartate aminotransferase
AUROC: area under the curve of the receiver operating characteristic
BA: basophils
BE: base excess
BG: blood glucose
BUN: blood urea nitrogen
Ca: calcium
CCA: complete case analysis
Cl: chloride
CP: complexity parameter
Cr: creatinine
DT: decision tree
ECI: Elixhauser Comorbidity Index
EHR: electronic health record
EO: eosinophils
FiO_2_: fraction of inspired oxygen
HCO _3_: bicarbonate
HCT: hematocrit
HD: hot deck
HGB: hemoglobin
ICU: intensive care unit
InfGain: information gain
K: potassium
Lac: lactate
LASSO: least absolute shrinkage and selection operator
LR: logistic regression
LT: laboratory test
LY: lymphocytes
MAR: missing at random
MCAR: missing completely at random
MCH: mean corpuscular hemoglobin
MCHC: mean corpuscular hemoglobin concentration
MCV: mean corpuscular volume
Mg: magnesium
MIMIC: Medical Information Mart for Intensive Care
ML: machine learning
MO: monocytes
Na: sodium
NE: neutrophils
PaO_2_: partial pressure of oxygen in the arterial blood
PCO_2_: partial pressure of carbon dioxide
Phos: phosphate
PLT: platelet count
PMM: predictive mean matching
PO_2_: partial pressure of oxygen
PT: prothrombin time
PTT: partial thromboplastin time
RBC: red blood cell
RDW: red cell distribution width
RF: random forest
SAPS-II: Simplified Acute Physiology Score II
TBil: total bilirubin
WBC: white blood cell
