# Supplementary material for: A New Insight Into Missing Data in Intensive Care Unit Patient Profiles: Observational Study
Source: JMIR Med Inform. 2019 Jan 8;7(1):e11605. doi: 10.2196/11605 (PMC6329436; doi:10.2196/11605)
Supplement: Multimedia Appendix 1 [file medinform_v7i1e11605_app1.pdf]

Multimedia Appendix 1: Detailed Results

Table S1 The association between indicators (missing=1 and not missing =0) and mortality flag (deceased=1, alive=0) using Phi coefficient.

	**In-hospital Mortality**			**30-days Mortality**		
**Indicator**	Day1	Day2	Day3	Day1	Day2	Day3
**ALT**	-0.14	-0.12	-0.12	-0.14	-0.12	-0.11
**ALK**	-0.14	-0.12	-0.11	-0.14	-0.12	-0.1
**pH**	-0.11	-0.13	-0.15	-0.08	-0.1	-0.11
**PCO**	-0.11	-0.16	-0.18	-0.08	-0.13	-0.15
**PO**	-0.11	-0.16	-0.18	-0.08	-0.13	-0.15
**BE**	-0.11	-0.16	-0.18	-0.08	-0.13	-0.15
**AST**	-0.14	-0.12	-0.12	-0.14	-0.12	-0.11
**Na**	0.01	0.03	0.04	0.01	0.02	0.05
**K**	0	0.01	0.01	-0.01	0	0.03
**Cl**	0.03	0.03	0.04	0.02	0.03	0.06
**HCO**	0.03	0.03	0.04	0.02	0.03	0.05
**AG**	-0.01	0	0.01	-0.02	0	0.01
**BG**	-0.02	0	0.01	-0.02	-0.01	0.02
**BUN**	0.04	0.04	0.06	0.04	0.04	0.07
**Cr**	0.04	0.05	0.06	0.04	0.04	0.07
**Ca**	-0.1	-0.04	-0.04	-0.12	-0.05	-0.04
**WBC**	0.03	0.04	0.06	0.02	0.04	0.06
**RBC**	0.02	0.03	0.04	0.02	0.03	0.05
**HGB**	0.03	0.03	0.05	0.02	0.03	0.05
**HCT**	0.04	0.05	0.05	0.03	0.05	0.06
**MCV**	0.02	0.03	0.04	0.02	0.03	0.05
**MCH**	0.02	0.03	0.04	0.02	0.03	0.05
**MCHC**	0.02	0.03	0.05	0.02	0.03	0.05

**RDW**	0.02	0.03	0.05	0.02	0.03	0.05
**PLT**	0.03	0.04	0.05	0.03	0.04	0.05
**NE**	-0.1	-0.07	-0.08	-0.11	-0.08	-0.07
**LY**	-0.1	-0.07	-0.08	-0.11	-0.08	-0.07
**MO**	-0.1	-0.07	-0.08	-0.11	-0.08	-0.07
**EO**	-0.1	-0.07	-0.08	-0.11	-0.08	-0.07
**BA**	-0.1	-0.07	-0.08	-0.11	-0.08	-0.07
**LAC**	-0.12	-0.15	-0.15	-0.1	-0.13	-0.12
**Mg**	-0.03	0.01	0.02	-0.05	0.01	0.03
**PTT**	-0.04	-0.09	-0.09	-0.04	-0.09	-0.09
**Phos**	-0.1	-0.05	-0.04	-0.12	-0.06	-0.04
**PT**	-0.04	-0.09	-0.08	-0.04	-0.09	-0.09
**TBil**	-0.14	-0.12	-0.12	-0.14	-0.12	-0.11

Table S2 Detailed results for predictor importance evaluation with regard to 30-day mortality. Numbers represent the ranking after aggregating the ranking results from the three different feature-selection methods.

**Day One**				**Day Two**				**Day Three**			
**Hot Deck**		**PMM**		**Hot Deck**		**PMM**		**Hot Deck**		**PMM**	
BUN	0.742936	BUN	0.762397	BUN	0.9007	AG	0.795419	RDW	0.693305	RDW	0.748997
RDW	0.682517	RDW	0.680087	RDW	0.851829	HCO3	0.783337	BUN	0.666667	BUN	0.666667
MCHC	0.677031	MCHC	0.668965	HCO3	0.655154	BUN	0.77677	HCO3	0.543871	HCO3	0.544964
AG	0.524767	AG	0.540484	AG	0.580852	BE	0.609532	BE	0.49406	BE	0.540542
I-Ca	0.475564	I-Ca	0.436429	MCHC	0.448053	RDW	0.608711	AG	0.449286	pH	0.488433
I-Phos	0.46459	Cr	0.43607	Cr	0.40508	I-PO2	0.58715	Cr	0.39854	AG	0.45042

			1		5		1		3		6
PO2	0.447435	HCO3	0.416741	Cl	0.382846	I-PCO2	0.585947	I-PCO2	0.375211	I-LAC	0.418716
HCO3	0.444744	PO2	0.404289	MCV	0.375821	I-BE	0.585592	I-PO2	0.374931	I-pH	0.40463
Cr	0.428755	MCV	0.386964	I-LAC	0.359897	Cl	0.53158	I-BE	0.374127	Cr	0.400008
I-LAC	0.38719	I-Phos	0.374431	Na	0.358051	PT	0.462085	PCO2	0.359771	Phos	0.387661
HGB	0.373706	PTT	0.353913	PTT	0.356926	LAC	0.461869	NE	0.357608	I-PCO2	0.387019
MCV	0.369112	HGB	0.342786	Phos	0.337663	Cr	0.451999	MCHC	0.331802	I-PO2	0.386739
LY	0.367866	pH	0.32767	PT	0.333779	PTT	0.424956	PT	0.328478	I-BE	0.385935
PTT	0.33424	LAC	0.320339	I-PO2	0.3335	Na	0.422474	LAC	0.289964	PCO2	0.367257
RBC	0.333482	BE	0.320299	I-PCO2	0.332814	Phos	0.419171	LY	0.287681	NE	0.360791
I-TBil	0.320728	I-LAC	0.318216	I-BE	0.332517	I-LAC	0.415475	pH	0.283643	MCV	0.351266
BG	0.319001	PCO2	0.316668	BE	0.290359	MCV	0.368343	MCV	0.281627	I-PTT	0.338352
I-ALT	0.315839	I-TBil	0.31277	I-Phos	0.284644	MCHC	0.363146	I-LAC	0.270327	LAC	0.331205
I-AST	0.314876	I-ALK	0.305529	I-TBil	0.283694	I-pH	0.352443	Phos	0.262758	MCHC	0.329817
PT	0.303447	I-ALT	0.303033	I-pH	0.273527	I-PT	0.338144	I-pH	0.239426	PT	0.329586
I-ALK	0.302031	I-AST	0.302074	I-PTT	0.261553	I-TBil	0.325466	BG	0.221239	I-PT	0.29994

LAC	0.277614	PT	0.296326	I-PT	0.258242	I-PTT	0.325362	I-PTT	0.221211	RBC	0.238642
Cl	0.276099	RBC	0.293754	PLT	0.252963	pH	0.312481	WBC	0.217481	I-Phos	0.237935
pH	0.273849	Phos	0.288846	I-Ca	0.248052	PCO2	0.307902	I-PT	0.214963	I-TBil	0.237811
PLT	0.273301	LY	0.285421	NE	0.247463	BG	0.301989	I-TBil	0.213362	BG	0.222807
I-PT	0.256103	ALK	0.284528	I-ALK	0.236376	I-ALK	0.30149	I-ALT	0.205211	WBC	0.219308
PCO2	0.255203	BG	0.282668	LAC	0.232278	PO2	0.297596	PTT	0.204495	I-ALT	0.21435
I-PTT	0.249578	PLT	0.279892	I-ALT	0.229095	PLT	0.29116	I-AST	0.202047	LY	0.212783
MCH	0.232707	NE	0.266646	I-AST	0.223172	I-ALT	0.285826	I-ALK	0.198413	I-AST	0.209672
Phos	0.218656	Cl	0.223353	BG	0.218129	TBil	0.283123	PLT	0.198322	PO2	0.208279
BE	0.208477	TBil	0.216197	HGB	0.208915	I-AST	0.281695	Cl	0.188406	PTT	0.206123
Na	0.207431	MCH	0.212788	PCO2	0.201961	LY	0.271601	PO2	0.180645	PLT	0.200699
I-NE	0.200687	I-NE	0.196231	WBC	0.201917	MCH	0.261553	I-Phos	0.167356	Cl	0.195587
I-MO	0.200687	I-MO	0.196231	Ca	0.193641	I-Phos	0.2614	I-Ca	0.165253	I-ALK	0.19224
I-EO	0.200687	I-EO	0.196231	BA	0.186936	Ca	0.242739	Mg	0.154738	Ca	0.185077
I-BA	0.200687	I-BA	0.196231	RBC	0.181905	WBC	0.242647	Na	0.146887	Mg	0.156356
I-LY	0.20060	I-LY	0.19615	pH	0.16804	I-Ca	0.20086	Ca	0.14404	Na	0.14953

	8		2		3		5		9		4
I-PCO2	0.187662	WBC	0.171699	HCT	0.16619	HGB	0.193331	TBil	0.140346	MCH	0.146659
I-PO2	0.187662	Ca	0.167312	LY	0.159886	RBC	0.188082	RBC	0.107089	I-Ca	0.144385
I-BE	0.187534	AST	0.162532	Mg	0.131304	ALK	0.184185	I-NE	0.097467	TBil	0.142264
NE	0.17315	I-PCO2	0.156981	TBil	0.126985	BA	0.169845	I-LY	0.097467	EO	0.142107
WBC	0.166962	I-PO2	0.156981	MCH	0.120035	AST	0.16688	I-MO	0.097467	BA	0.142031
Ca	0.163823	I-BE	0.156853	I-NE	0.103639	Mg	0.159173	I-EO	0.097467	I-NE	0.099961
I-pH	0.159875	BA	0.149717	I-LY	0.103639	NE	0.150139	I-BA	0.097467	I-LY	0.099961
I-Mg	0.14009	Na	0.146076	I-MO	0.103639	HCT	0.117954	K	0.089782	I-MO	0.099961
ALK	0.13104	I-pH	0.144973	I-EO	0.103639	EO	0.114959	AST	0.084185	I-EO	0.099961
TBil	0.111702	I-Mg	0.11108	I-BA	0.103639	I-NE	0.113355	ALK	0.082093	I-BA	0.099961
EO	0.109363	I-BG	0.106693	ALK	0.094855	I-LY	0.113355	I-Mg	0.070701	I-RDW	0.094193
BA	0.108785	EO	0.106064	MO	0.09224	I-MO	0.113355	BA	0.069968	K	0.092206
I-BG	0.103206	I-AG	0.10267	K	0.075067	I-EO	0.113355	I-Cl	0.069821	I-RBC	0.08586
I-AG	0.098421	I-PT	0.092205	EO	0.063887	I-BA	0.113355	I-Cr	0.069027	I-MCV	0.08586
AST	0.085218	I-PTT	0.08084	I-Mg	0.063662	MO	0.108044	I-BUN	0.06785	I-MCH	0.08586

I-K	0.085122	MO	0.062942	PO2	0.055418	K	0.103865	I-BG	0.065149	ALK	0.084326
ALT	0.061017	ALT	0.055905	I-HCT	0.055202	ALT	0.078095	MO	0.056612	AST	0.081458
Mg	0.052026	Mg	0.044178	I-MCHC	0.045723	I-RDW	0.077468	I-HCT	0.054567	I-MCHC	0.080782
I-BUN	0.048802	HCT	0.044135	I-RBC	0.045597	I-MCHC	0.076733	I-PLT	0.052654	I-Mg	0.065196
HCT	0.047372	I-PLT	0.035278	I-MCV	0.045597	I-RBC	0.076601	I-HCO3	0.050589	I-Cr	0.059682
I-Cr	0.045343	K	0.034445	I-MCH	0.045597	I-MCV	0.076601	I-K	0.049123	I-BUN	0.058504
MO	0.037529	I-K	0.034074	I-RDW	0.045242	I-MCH	0.076601	EO	0.048483	I-WBC	0.055585
K	0.0359	I-HGB	0.033298	AST	0.04516	I-HGB	0.069039	MCH	0.048214	I-HGB	0.055267
I-Na	0.028494	I-MCV	0.033014	I-BUN	0.031937	I-HCT	0.063231	I-WBC	0.04705	MO	0.055048
I-Cl	0.026307	I-MCH	0.033014	I-PLT	0.03054	I-PLT	0.057216	I-Na	0.046679	I-HCT	0.054935
I-PLT	0.023585	I-MCHC	0.03284	ALT	0.030451	I-WBC	0.055654	ALT	0.045415	I-PLT	0.048878
I-HCO3	0.023289	I-RDW	0.032836	I-WBC	0.028815	I-Cr	0.043887	I-HGB	0.039608	I-Cl	0.044026
I-WBC	0.015726	I-RBC	0.032737	I-Cr	0.028358	I-BUN	0.041846	I-RDW	0.035126	I-K	0.043233
I-HCT	0.012493	I-WBC	0.028194	I-HGB	0.027524	I-Cl	0.034206	I-MCHC	0.032941	I-Na	0.04292
I-HGB	0.011658	I-HCT	0.023648	I-K	0.027506	I-HCO3	0.033703	I-RBC	0.032401	I-HCO3	0.041208
I-MCV	0.01137	I-Cr	0.01678	I-AG	0.02130	I-Na	0.03139	I-MCV	0.03240	I-BG	0.03874

	3				7		3		1		8
I-MCH	0.011373	I-BUN	0.016679	I-BG	0.020366	I-Mg	0.022961	I-MCH	0.032401	I-AG	0.030697
I-MCHC	0.011198	I-Na	0.014031	I-Cl	0.019271	I-BG	0.017067	HGB	0.0278	HGB	0.030518
I-RDW	0.011194	I-Cl	0.007722	I-HCO3	0.01926	I-AG	0.015687	I-AG	0.025759	HCT	0.02312
I-RBC	0.011095	I-HCO3	0.005457	I-Na	0.013649	I-K	0.013539	HCT	0.020315	ALT	0

Table S3 Detailed results for predictor importance evaluation with regard to in-hospital mortality. Numbers represent the ranking after aggregating the ranking results from the three different feature-selection methods.

**Day One**				**Day Two**				**Day Three**			
**Hot Deck**		**PMM**		**Hot Deck**		**PMM**		**Hot Deck**		**PMM**	
BUN	0.792686	BUN	0.825715	BUN	0.871227	BUN	1	BE	0.66376	RDW	0.75246
AG	0.66198	AG	0.668918	AG	0.856826	RDW	0.711852	BUN	0.640534	BUN	0.635729
RDW	0.599006	RDW	0.573188	RDW	0.810929	HCO3	0.684191	HCO3	0.626034	BE	0.633926
HCO3	0.590773	HCO3	0.531746	HCO3	0.802246	AG	0.664339	RDW	0.61847	HCO3	0.62367
MCHC	0.584486	MCHC	0.507343	I-PO2	0.594496	BE	0.528778	I-BE	0.587481	I-BE	0.595553
BG	0.53102	PCO2	0.489483	I-PCO2	0.593258	MCHC	0.503805	I-PCO2	0.587166	I-PCO2	0.595238
PO2	0.521196	Cr	0.480181	I-BE	0.592893	PT	0.453111	I-PO2	0.586851	I-PO2	0.594924
Cr	0.487362	BE	0.452599	I-LAC	0.548438	Cl	0.429405	pH	0.515275	pH	0.556242

MCV	0.417346	I-LAC	0.436382	Cl	0.529452	I-LAC	0.425279	AG	0.494856	Phos	0.494694
I-LAC	0.411539	LAC	0.415773	PTT	0.511771	Cr	0.395266	I-LAC	0.489909	AG	0.492864
I-ALT	0.387559	HGB	0.414263	Phos	0.497639	I-PO2	0.382404	PCO2	0.438274	I-pH	0.470007
I-AST	0.387498	pH	0.402466	MCHC	0.485717	I-PCO2	0.381737	I-pH	0.437812	I-LAC	0.469215
I-ALK	0.385754	I-TBil	0.399363	Na	0.473093	I-BE	0.381448	Cr	0.416895	Cr	0.415249
I-TBil	0.384603	I-Ca	0.395278	Cr	0.461062	PTT	0.357339	Phos	0.355405	LAC	0.396136
I-Phos	0.381937	I-ALT	0.376004	I-pH	0.460122	Phos	0.352738	PT	0.328079	NE	0.338372
I-Ca	0.380717	I-AST	0.375944	Ca	0.422747	Na	0.345109	I-PTT	0.311758	PT	0.326491
LY	0.373098	LY	0.375163	PT	0.386761	I-PT	0.333936	NE	0.304336	LY	0.319146
PTT	0.36056	I-ALK	0.366346	PLT	0.371176	BG	0.320947	LAC	0.290642	MCV	0.314868
PLT	0.359362	RBC	0.36628	BE	0.365499	I-pH	0.317841	MCV	0.286645	PCO2	0.304013
Phos	0.358443	Phos	0.360009	I-PT	0.362492	LAC	0.307212	I-PT	0.280568	MCHC	0.297485
HGB	0.353079	BG	0.359947	BG	0.356136	PO2	0.295944	I-TBil	0.276302	RBC	0.280764
LAC	0.345373	PO2	0.333154	LAC	0.343797	MCV	0.293408	I-ALT	0.273609	I-AST	0.274608
I-PCO2	0.340782	PLT	0.332843	I-PTT	0.342374	HGB	0.289974	I-AST	0.269524	I-TBil	0.274146
I-PO2	0.34078	MCV	0.33067	MCV	0.34119	PCO2	0.28705	MCHC	0.25742	I-ALT	0.27095

	2		6		8		6		5		7
I-BE	0.340621	PT	0.324238	RBC	0.30348	I-PTT	0.286421	BG	0.256086	I-ALK	0.262906
PCO2	0.336776	I-Phos	0.324092	I-TBil	0.300585	NE	0.283794	PLT	0.254084	I-PTT	0.260668
PT	0.328778	I-PCO2	0.31347	PCO2	0.300358	I-TBil	0.282065	I-ALK	0.25055	I-PT	0.25894
RBC	0.313951	I-PO2	0.31347	I-ALK	0.295056	LY	0.264846	WBC	0.240404	BG	0.254835
pH	0.312077	I-BE	0.313312	I-ALT	0.293048	TBil	0.25852	RBC	0.229375	PLT	0.253083
BE	0.301225	PTT	0.313247	I-AST	0.288134	RBC	0.257641	Cl	0.224759	WBC	0.239337
I-pH	0.293267	I-pH	0.312705	HGB	0.287403	MCH	0.256053	I-Ca	0.220419	Cl	0.230215
NE	0.291994	ALK	0.258992	I-Phos	0.278502	pH	0.254797	I-Phos	0.211873	PTT	0.201343
Cl	0.282029	Na	0.228743	PO2	0.247892	I-ALK	0.245638	PTT	0.202375	Mg	0.188997
MCH	0.259909	TBil	0.227722	WBC	0.245929	I-ALT	0.23875	Mg	0.190069	PO2	0.173802
WBC	0.225897	AST	0.226631	LY	0.230429	I-AST	0.237073	Ca	0.182171	BA	0.164709
I-NE	0.218566	WBC	0.221189	pH	0.21219	BA	0.229466	LY	0.163745	Ca	0.163818
I-MO	0.218566	I-NE	0.214159	MCH	0.204618	PLT	0.227216	PO2	0.160079	MO	0.161944
I-EO	0.218566	I-MO	0.214159	I-Ca	0.202655	WBC	0.193095	Na	0.150253	MCH	0.155433
I-BA	0.218566	I-EO	0.214159	HCT	0.195806	ALK	0.187838	TBil	0.139763	Na	0.149719

I-LY	0.218487	I-BA	0.214159	NE	0.194221	HCT	0.180302	MCH	0.131544	TBil	0.139005
Ca	0.209441	I-LY	0.214082	BA	0.189756	Ca	0.171374	K	0.117619	EO	0.136577
Na	0.195981	Ca	0.211277	Mg	0.159471	I-Phos	0.14342	I-NE	0.114392	K	0.117024
I-PT	0.185589	NE	0.204053	TBil	0.136629	MO	0.142681	I-LY	0.114392	I-Phos	0.115488
I-PTT	0.183623	BA	0.18897	I-NE	0.125957	Mg	0.134089	I-MO	0.114392	I-NE	0.113881
TBil	0.138903	EO	0.17213	I-LY	0.125957	AST	0.119857	I-EO	0.114392	I-LY	0.113881
AST	0.134067	MCH	0.170869	I-MO	0.125957	I-NE	0.112565	I-BA	0.114392	I-MO	0.113881
EO	0.127119	I-PT	0.170498	I-EO	0.125957	I-LY	0.112565	BA	0.110613	I-EO	0.113881
ALK	0.110338	I-PTT	0.160825	I-BA	0.125957	I-MO	0.112565	AST	0.108952	I-BA	0.113881
BA	0.108381	Cl	0.154443	K	0.096208	I-EO	0.112565	MO	0.083459	AST	0.112826
MO	0.099866	ALT	0.098121	AST	0.079471	I-BA	0.112565	EO	0.074052	I-Ca	0.088959
I-Mg	0.09522	I-BG	0.095042	MO	0.073539	I-Ca	0.097019	I-Mg	0.072413	ALK	0.066881
ALT	0.086944	I-AG	0.081084	EO	0.059083	EO	0.090394	ALK	0.067309	I-RDW	0.059171
Mg	0.05808	I-Mg	0.06207	ALK	0.050719	I-BG	0.077374	ALT	0.06125	I-MCHC	0.057317
K	0.055639	I-K	0.054604	I-MCHC	0.049679	I-AG	0.070263	I-WBC	0.047277	I-RBC	0.056806
I-HCT	0.03807	K	0.05459	I-RBC	0.04954	K	0.06847	I-Cr	0.04698	I-MCV	0.05680

	4		8		3		7		7		6
I-AG	0.03646	Mg	0.041903	I-MCV	0.049543	I-K	0.064423	I-BUN	0.043822	I-MCH	0.056806
I-BG	0.034724	I-RBC	0.039129	I-MCH	0.049543	ALT	0.043276	I-PLT	0.040581	I-Cr	0.050183
I-BUN	0.028065	I-MCV	0.039069	I-HCT	0.047944	I-HCT	0.036985	I-HCT	0.03936	I-PLT	0.047133
I-K	0.027928	I-MCH	0.039069	I-HGB	0.047702	I-RDW	0.03124	I-RDW	0.039224	I-WBC	0.047106
I-Cr	0.027837	I-MCHC	0.038744	I-RDW	0.046801	I-PLT	0.029356	I-MCHC	0.037362	I-BUN	0.047027
HCT	0.024159	I-HCT	0.036842	ALT	0.0414	I-WBC	0.027678	I-RBC	0.036849	I-HCT	0.045927
I-PLT	0.022738	I-RDW	0.036652	I-BUN	0.040751	I-Cr	0.027393	I-MCV	0.036849	I-HGB	0.038626
I-WBC	0.019027	I-Cr	0.0327	I-PLT	0.03946	I-BUN	0.026789	I-MCH	0.036849	I-Cl	0.034929
I-RDW	0.018714	I-PLT	0.03036	I-Cr	0.039221	I-MCHC	0.023899	I-HGB	0.035416	I-HCO3	0.034152
I-HGB	0.016144	I-BUN	0.030341	I-WBC	0.034229	I-RBC	0.023775	I-Cl	0.031698	I-AG	0.030166
I-RBC	0.015815	I-HCO3	0.021465	I-Cl	0.02632	I-MCV	0.023775	I-HCO3	0.03092	I-K	0.027199
I-MCV	0.015754	I-Cl	0.021105	I-HCO3	0.025802	I-MCH	0.023775	HGB	0.027294	HGB	0.027109
I-MCH	0.015754	I-WBC	0.018852	I-Na	0.020485	I-HGB	0.022524	I-Na	0.023554	I-Na	0.026809
I-MCHC	0.015422	HCT	0.018361	I-Mg	0.019784	I-HCO3	0.022448	HCT	0.020428	I-Mg	0.024299
I-HCO3	0.01364	I-Na	0.016962	I-K	0.005176	I-Mg	0.020097	I-BG	0.007874	HCT	0.020301

I-Cl	0.013272	I-HGB	0.016188	I-AG	0.00514	I-Cl	0.018554	I-AG	0.003873	ALT	0.017294
I-Na	0.009183	MO	0.008405	I-BG	0.003625	I-Na	0.016975	I-K	0.003757	I-BG	0.010681

Table S4 Detailed AUROC values for all three days models for 30-days mortality.

		**Day1**		**Day2**		**Day3**	
		**AUROC**	**AUROC-SD**	**AUROC**	**AUROC-SD**	**AUROC**	**AUROC-SD**
**Logistic Regression**							
	Indicator Only	0.683639	0.012024	0.662923	0.010076	0.658562	0.016731
	HD	0.764109	0.007059	0.742167	0.015646	0.734406	0.009016
	HD + Indicator	0.785046	0.008317	0.76738	0.013904	0.761211	0.01254
	PMM	0.765781	0.00824	0.749135	0.013908	0.733725	0.012114
	PMM + Indicator	0.786277	0.010084	0.772205	0.012432	0.760894	0.013726
	SAPS II	0.781272	0.010389	0.734669	0.016996	0.704888	0.014114
	SAPS II +Indicator	0.804469	0.010902	0.758655	0.014826	0.738983	0.013721
**Decision Tree**							
	Indicator Only	0.650103	0.030717	0.626416	0.013378	0.629829	0.029985
	HD	0.710146	0.008507	0.686409	0.018551	0.664421	0.023127
	HD + Indicator	0.721343	0.009624	0.699798	0.017404	0.673743	0.010208
	PMM	0.707519	0.010437	0.683301	0.024034	0.660748	0.024769
	PMM + Indicator	0.714665	0.016703	0.695593	0.022077	0.676769	0.023449
	SAPS II	0.781272	0.010389	0.64003	0.020941	0.631917	0.014237
	SAPS II +Indicator	0.804469	0.010902	0.733759	0.019449	0.716765	0.015101
**Random Forest**							
	Indicator Only	0.505464	0.006078	0.514214	0.011948	0.530299	0.009883
	HD	0.773757	0.008081	0.7451	0.011768	0.72928	0.007846
	HD + Indicator	0.792436	0.008205	0.766697	0.011666	0.754279	0.010198

	PMM	0.778668	0.009603	0.757371	0.00969	0.741238	0.014159
	PMM + Indicator	0.790357	0.01027	0.77025	0.010431	0.751982	0.011704
	SAPS II	0.598363	0.007601	0.582176	0.013028	0.581908	0.010418
	SAPS II +Indicator	0.702174	0.009598	0.669676	0.016399	0.662755	0.014661

Table S5 Detailed AUROC values for all three days models for in-hospital mortality.

		**Day1**		**Day2**		**Day3**	
		**AUROC**	**AUROC-SD**	**AUROC**	**AUROC-SD**	**AUROC**	**AUROC-SD**
**Logistic Regression**							
	Indicator Only	0.714672	0.017386	0.691756	0.012501	0.692288	0.015078
	HD	0.764219	0.018237	0.741566	0.01794	0.732094	0.0221
	HD + Indicator	0.798866	0.01876	0.779967	0.01663	0.774248	0.013158
	PMM	0.764211	0.019857	0.747549	0.016608	0.73011	0.018976
	PMM + Indicator	0.798734	0.018334	0.783444	0.015014	0.772708	0.012686
	SAPS II	0.787666	0.014567	0.733107	0.011018	0.697324	0.017352
	SAPS II +Indicator	0.817011	0.014251	0.768525	0.013978	0.751957	0.013731
**Decision Tree**							
	Indicator Only	0.617608	0.084701	0.645272	0.010946	0.518569	0.010091
	HD	0.712456	0.016019	0.685595	0.025948	0.732607	0.018908
	HD + Indicator	0.733814	0.021929	0.718591	0.014642	0.768959	0.015584
	PMM	0.712825	0.014675	0.692577	0.013314	0.740328	0.019537
	PMM + Indicator	0.731189	0.017454	0.72262	0.023738	0.765453	0.014443
	SAPS II	0.661025	0.021642	0.64404	0.016882	0.545543	0.009066
	SAPS II +Indicator	0.785751	0.016167	0.740022	0.012199	0.662513	0.015397
**Random Forest**							
	Indicator Only	0.506492	0.002377	0.511513	0.004844	0.518569	0.010091
	HD	0.774549	0.01358	0.748636	0.021041	0.732607	0.018908
	HD + Indicator	0.802737	0.013752	0.778569	0.018496	0.768959	0.015584

	PMM	0.787579	0.015326	0.759913	0.019799	0.740328	0.019537
	PMM + Indicator	0.802973	0.01194	0.780929	0.016078	0.765453	0.014443
	SAPS II	0.579601	0.010443	0.556754	0.013962	0.545543	0.009066
	SAPS II +Indicator	0.689969	0.015505	0.658816	0.017864	0.662513	0.015397
